# Surrogates of Muscle Mass on Cardiac MRI Correlate with Exercise Capacity in Patients with Fontan Circulation

**DOI:** 10.3390/jcm12072689

**Published:** 2023-04-04

**Authors:** Kevin L. Smith, Emile B. Gordon, Megan E. Gunsaulus, Adam Christopher, Laura J. Olivieri, Sameh S. Tadros, Tyler Harris, Anita P. Saraf, Jacqueline Kreutzer, Brian Feingold, Tarek Alsaied

**Affiliations:** 1 Department of Pediatrics, UPMC Children’s Hospital of Pittsburgh, University of Pittsburgh School of Medicine, Pittsburgh, PA 15224, USA; 2 Department of Radiology, UPMC Children’s Hospital of Pittsburgh, University of Pittsburgh School of Medicine, Pittsburgh, PA 15224, USA; 3 Department of Pediatric Cardiology, UPMC Children’s Hospital of Pittsburgh, University of Pittsburgh School of Medicine, Pittsburgh, PA 15224, USA

**Keywords:** cardiac MRI, Fontan, sarcopenia, exercise stress test, cardiorespiratory fitness, muscle mass

## Abstract

Background: Sarcopenia is an increasingly recognized marker of frailty in cardiac patients. Patients with a history of congenital heart disease and Fontan procedure have a higher risk of developing progressive muscle wasting. Our objective was to determine if we could use routine cardiac MRI (CMR) for the surveillance of muscle wasting. Methods: A retrospective study of all Fontan patients (n = 75) was conducted at our institution, with CMR performed from 2010 to 2022 and exercise stress testing performed within 12 months (4.3 ± 4.2 months). The skeletal muscle area (SMA) for the posterior paraspinal and anterior thoracic muscles were traced and indexed for body surface area (BSA). Patients were stratified by percentile into the upper and lower quartiles, and the two groups were compared. Multivariable regression was performed to control for sex and age. Results: There was a significant positive association of both anterior (r = 0.34, *p* = 0.039) and paraspinal (r = 0.43, *p* = 0.007) SMA to peak VO_2_. Similarly, paraspinal but not anterior SMA was negatively associated with the V_E_/V_CO2_ (r = –0.45, *p* = 0.006). The upper quartile group had significantly more males (18/19 vs. 8/20; *p* = 0.0003) and demonstrated a significantly higher peak VO_2_ (32.2 ± 8.5 vs. 23.8 ± 4.7, *p* = 0.009), a higher peak RER (1.2 ± 0.1 vs. 1.1 ± 0.04, *p* = 0.007), and a significantly lower V_E_/V_CO2_ (32.9 ± 3.6 vs. 40.2 ± 6.2, *p* = 0.006) compared to the lowest quartile. The association of SMA to VO_2_ peak and V_E_/V_CO2_ was redemonstrated after controlling for sex and age. Conclusion: Thoracic skeletal muscle area may be an effective surrogate of muscle mass and is correlated to several measures of cardiorespiratory fitness post-Fontan. CMR would be an effective tool for the surveillance of sarcopenia in post-Fontan patients given its accessibility and routine use in these patients.

## 1. Introduction

The Fontan operation is the final palliative procedure for single-ventricle hearts, and the procedure redirects systemic venous return to pulmonary arteries without a sub-pulmonary pumping chamber [1]. Despite the improvement in outcomes, patients may still face numerous complications such as heart failure stemming from systolic or diastolic ventricular dysfunction [2,3], protein-losing enteropathy [4,5], arrhythmias [2], muscle wasting [3,6], and impaired exercise capacity [7,8]. Fontan patients are less physically active compared to their healthy counterparts because of various cardiopulmonary factors [9]. Physiological changes, including but not limited to increased dead space ventilation, reduced vital capacity, high pulmonary arterial wedge pressure, and an inability to maintain stroke volume during exercise, contribute to hypercapnia and subsequent inefficient cardiorespiratory function during exercise [10,11]. Exercise intolerance serves as a prognostic marker in congenital heart disease patients; thus, a better understanding of exercise stress testing and its relationship to disease progression can be a valuable tool in disease surveillance [12,13,14,15].

In adult patients with Fontan circulation, muscle wasting is a common complication [16,17] that is associated with adverse events in other chronic medical conditions such as cancer [18,19] and heart failure [20,21]. In children, the Fontan operation has been associated with abnormalities in body composition, bone strength, and growth [6,22,23]. Assessment of the abdominal skeletal muscle at the third lumbar vertebra (L3) has been common practice to define sarcopenia in non-Fontan patients [24,25]. Sarcopenia has also been assessed by measuring abdominal muscle mass in Fontan patients on abdominal magnetic resonance imaging (MRI) performed initially to evaluate for liver disease [26]. Lower skeletal muscle mass seen on abdominal MRI has been associated with decreased cardiorespiratory fitness; however, abdominal MRI is not routinely performed in Fontan patients [26]. Cardiac MRIs (CMR), on the other hand, are routinely used post-Fontan and provide important diagnostic information. The objective of this study is to assess the feasibility and reproducibility of determining skeletal muscle mass via thoracic muscle groups seen on routine CMR and assess the correlation of muscle mass with exercise capacity, specifically peak VO_2_, V_E_/V_CO2_, and respiratory exchange ratio (RER).

## 2. Materials and Methods

### 2.1. Study Population

This was a retrospective study conducted at the University of Pittsburgh Medical Center, Children’s Hospital of Pittsburgh. All patients with a history of Fontan surgery who had a CMR were included in this study. Patients who did not have adequate CMR images to measure the thoracic muscle mass or sufficient exercise stress testing data were excluded. A total of 10 patients was excluded based on this criterion, making the total study population 75 patients.

### 2.2. Data Collection

Data for baseline and demographic characteristics, imaging, and cardiopulmonary exercise testing were collected from patient charts. The data dictionary and definitions from the FORCE registry (Fontan Outcome Registry using CMR Examinations) were used [27]. Research Electronic Data Capture (REDCap) was used to store the data for this study. This study was approved by the University of Pittsburgh Institutional Review Board and was conducted in compliance with the Health Insurance Portability and Accountability Act. The requirement for informed consent was waived due to the retrospective nature of the study.

### 2.3. CMR Data Analysis

CMR studies were performed by using 1.5 Tesla scanners (GE Medical Systems, Milwaukee, WI, USA or Siemens AG, Munich, Germany). Briefly, ventricular assessment was performed via an electrocardiographically gated, balanced steady-state free precession (bSSFP) cine CMR in vertical and horizontal ventricular long-axis planes and a stack of slices in a ventricular short-axis plane encompassing the atrioventricular junction through the cardiac apex. Retrospectively cardiac-gated, through-plane phase-contrast flow measurements were obtained in the branch pulmonary arteries and venae cavae. If a patient had multiple CMR studies, the most recent study was used for analysis. The mean time period from Fontan operation and CMR was 14.1 ± 7.7 years. Ventricular volumes and function were measured by manually tracing the endocardial and epicardial borders on each short-axis bSSFP cine slice at end-diastole (maximal volume) and end-systole (minimal volume). Analysis was performed by using commercially available software (Cvi-42, Circle Cardiovascular Imaging Inc., Calgary, AB, Canada) and IBM SSPS Statistics (Version 27, Armonk, NY, USA: IBM Corp). All CMR exams were reanalyzed by a single pediatric cardiologist with clinical experience interpreting CMR studies in the single ventricle population. Ventricular contours were redrawn [28].

### 2.4. Muscle Measurements

**Figure 1:**
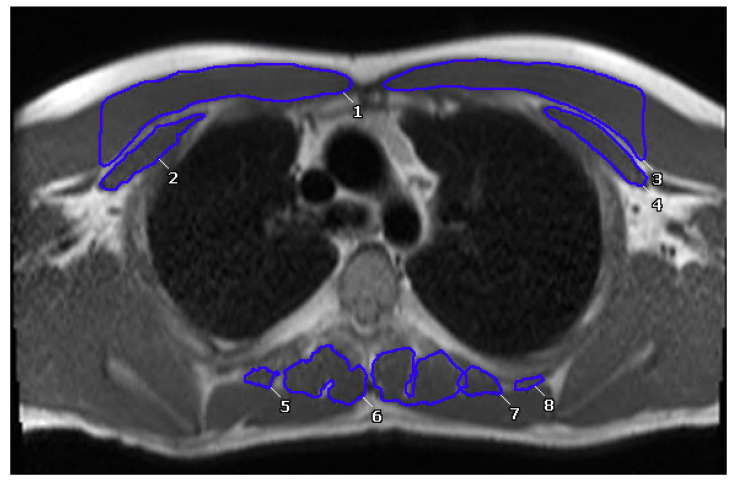
Segmentation of anterior and posterior paraspinal muscles at the level of the carina. Numbers seen on scan signify different hand traced muscles to quantify total muscle area of anterior and posterior muscles.

Muscle measurements were obtained by analyzing each patient’s most recent CMR. The skeletal muscle area (SMA) of the patients was assessed in the axial view on bright blood bSSFP static images at the level of Carina with offline analysis software (Cvi-42, Circle Cardiovascular Imaging Inc., Calgary, AB, Canada). Measurements of muscle area were conducted in images in static SSFP axial stack. The pectoralis major and minor muscles were traced for the assessment of anterior muscle area, while the paraspinal muscles were traced for posterior muscle area at the level of T4 (Figure 1) [29]. Due to the large variation in height and body habitus of our cohort, SMA, which is reported in cm^2^, was indexed by body surface area (BSA) by using the Mosteller calculation [30]. BSA-indexed SMA was reported as cm^2^/m^2^. Due to a lack of standardized values that matched our baseline characteristics in a non-Fontan population, we divided patients into high- and low-muscle groups, with high being the upper 25th percentile and low being the lower 25th percentile for BSA-indexed SMA.

### 2.5. Cardiopulmonary Exercise Testing

Patients underwent CPET by using a treadmill, as per the Bruce protocol. Gas exchange was analyzed at rest, during exercise, and during recovery to determine measures of oxygen uptake (VO_2_). Since peak VO_2_ is influenced by age, sex, and body weight, the percent of predicted peak VO_2_ value (% predicted VO_2_) was used due to the wide age range in this study [31]. Cardiopulmonary exercise stress testing data were collected within 12 months (4.3 ± 4.2 months) of the most recent CMR.

### 2.6. Statistical Analyses

Categorical variables were reported as counts and percentages, while continuous variables were expressed as mean ± standard deviation. Comparison between two continuous variables was performed by using t-testing or Mann–Whitney U for group sizes less than 30 subjects, while Chi-square tests were used for comparing categorical variables. Comparison between multiple continuous variables was performed by using Kruskal–Wallis testing, accounting for uneven distribution. Correlational testing was performed by using the Pearson correlation test. Multivariate analysis to adjust for sex and age was expressed as a parameter estimate ± standard deviation. Due to the sample size, muscle mass measurements were adjusted for one covariate at a time, and the results were reported. Cohen’s d and Hedges’ g tests were used to calculate effect sizes. Cohen’s d test was used for groups with sample sizes > 20 or groups with similar sample sizes. Hedges’ g test was used for sample sizes < 20 or groups that differed greatly in size. Cohen’s d test is reported as *d,* and Hedges’ g test is reported as *g*. To account for observer variability in muscle area tracing, there was retracing of ten random scans by another observer, and a comparison between SMA measurements of anterior and posterior muscle area was performed. A *p*-value of <0.05 was considered statistically significant. Interclass correlation was reported. Statistical analysis was conducted by using JMP Pro, Version 16.2.0.

## 3. Results

Our cohort consisted of seventy-five patients with Fontan circulation. The mean age at CMR was 19.1 ± 8.6 years and 49 (65%) males. In total, 1 patient had an atriopulmonary Fontan (1.3%), 49 patients (65%) had an extracardiac conduit Fontan, 23 (31%) had a lateral tunnel Fontan, and 2 (2.6%) were unknown. No reported deaths or transplant. Additional baseline characteristics are listed in Table 1 and Table 2.

### 3.1. Anterior and Paraspinal Muscle Index to BSA in the Fontan Population

The mean BSA-indexed anterior muscle and paraspinal muscle area was 20.8 ± 5.7 cm^2^/m^2^ and 6.5 ± 1.7 cm^2^/m^2^, respectively. Following exclusion of ineligible patients, our study cohort consisted of 75 patients with CMR (Table 1). In the rest of the patients, there was artifact at the level of the carina precluding accurate measurements. Combined BSA-indexed muscle area was 27.3 ± 6.6 cm^2^/m^2^. BSA-indexed anterior muscle, paraspinal muscle, and combined muscle areas were all significantly larger in male patients than female patients (BSA-indexed anterior muscle area: 22.4 ± 6.0 cm^2^/m^2^ vs. 17.8 ± 3.3 cm^2^/m^2^, *p =* 0.0004; BSA-indexed paraspinal muscle area: 6.9 ± 1.8 cm^2^/m^2^ vs. 5.9 ± 1.3 cm^2^/m^2^, *p =* 0.0105; BSA-indexed combined muscle area: 29.3 ± 7.0 cm^2^/m^2^ vs. 23.6 ± 3.8 cm^2^/m^2^, *p =* 0.0002). The differences between males and females are summarized in Table 3.

### 3.2. Intra-Observer and Inter-Observer Reliability:

**Figure 2:**
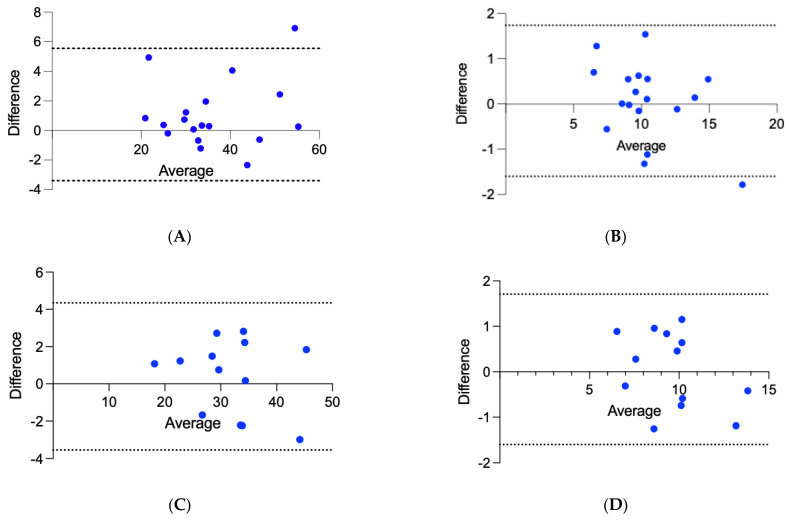
Bland Altman plots for inter-observer measurements of (**A**) anterior and (**B**) paraspinal muscle areas and intra-observer measurements of (**C**) anterior and (**D**) paraspinal muscle areas. Central solid line is representative of the bias, with dotted lines being representative of 95% limits of agreement (**A**) 1.081 (95% CI, −3.4–5.6), (**B**) 0.07 (95% CI, −1.6–1.7), (**C**) 0.40 (95% CI, −3.5–4.4), and (**D**) 0.10 (95% CI, −1.6–1.7), respectively.

There was good intra-observer reliability for measurements of anterior and paraspinal SMA on CMR of 0.97 (95% CI, 0.89–0.99) and 0.93 (95% CI, 0.79–0.98). In Inter-observer reliability, there was good intra-class correlation for repeated measurements of anterior and paraspinal SMA on CMR of 0.98 (95% CI, 0.94–0.99) and 0.96 (95% CI, 0.90–0.99), respectively. The Bland Altman plot for measurements can be seen in Figure 2.

### 3.3. Comparison between High- and Low-Muscle Groups

Between low- and high-muscle groups, both cohorts were similar in age (17.2 ± 7.0 vs. 17.8 ± 7.1, *p* = 0.499, *d* = 0.10) with a significant difference in BMI (low: 25.1 ± 5.3 vs. high: 21.3 ± 4.9, *p =* 0.027, *d* = −0.76) and sex distribution (Low-muscle group: Males 40%, Females 60% vs. High-muscle group: Males 95%, Females 5%, *p =* 0.0003). Further characteristics are shown in Table 4.

### 3.4. Correlation of Muscle Mass with Exercise Capacity and Other Clinical Parameters

**Figure 3:**
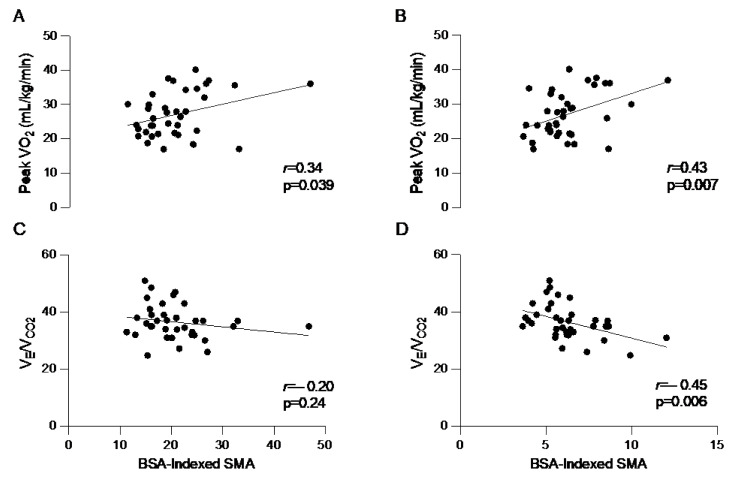
Correlation between BSA-indexed anterior SMA with (**A**) peak VO_2_, (**C**) V_E_/V_CO2_ and BSA-indexed paraspinal SMA with (**B**) peak VO_2_ and (**D**) V_E_/V_CO2_. VO_2_—oxygen consumption; V_E_/V_CO2_—minute ventilation/carbon dioxide production; BSA—body surface area; SMA—skeletal muscle area. Pearson correlation testing was performed.

BSA-indexed paraspinal and anterior muscle have a significant positive correlation with peak VO_2_ (r = 0.43, *p* = 0.0070 and r = 0.34, *p* = 0.0389). BSA-indexed paraspinal muscle area is negatively associated with V_E_/V_CO2_ (r = −0.45, *p* = 0.0060). Further characteristics are summarized on Figure 3 and Table 4.

**Figure 4:**
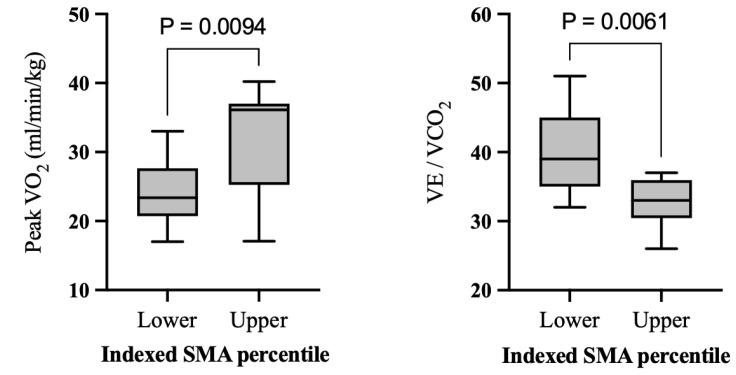
Significant differences in average peak VO_2_ and V_E_/V_CO2_ in low- and high-muscle groups. Data presented in box and whisker plots. Bars represent upper and lower quartiles. Low-muscle peak VO_2_ mean and SD is 23.8 ± 4.7 mL/kg/min (1st quartile: 20.7, median: 23.4, 3rd quartile: 27.6). High-muscle peak VO_2_ mean and SD is 32.2 ± 8.5 mL/kg/min (1st quartile: 25.2, median: 36.1, 3rd quartile: 37). Low-muscle VE/VCO_2_ mean and SD is 40.2 ± 6.2 (1st quartile: 35, median: 39, 3rd quartile: 45). High-muscle VE/VCO_2_ mean and SD is 32.9 ± 3.6 (1st quartile: 30.5, median: 33, 3rd quartile 36).

When conducting analysis based on high- and low-muscle groups, the high-muscle group had significantly higher peak VO_2_ (32.2 ± 8.5 vs. 23.8 ± 4.7, *p =* 0.009, g = 1.22), a higher peak RER (1.2 ± 0.1 vs. 1.1 ± 0.04, *p =* 0.007, g = 1.47) and lower V_E_/V_CO2_ (32.9 ± 3.6 vs. 40.2 ± 6.2, *p =* 0.006, g = −1.34) (Table 5 and Figure 4). When ventricular morphology was analyzed, the systemic right, left, or mixed ventricle did not significantly differ in any exercise parameters including peak VO_2_, RER, and V_E_/V_CO2_ (Supplemental Table S1).

Sex-specific analysis showed males had significantly higher % predicted peak VO_2_ (74.9 ± 9.9 vs. 60.3 ± 14.1, *p =* 0.002)

In multivariate analysis, BSA-indexed paraspinal muscle area was significantly associated with peak VO_2_ when adjusted for age (parameter estimate ± standard error, 1.7 ± 0.6 per 1cm^2^/m^2^, *p =* 0.008) and sex (1.5 ± 0.6 per 1cm^2^/m^2^, *p =* 0.019). Similarly, BSA-indexed anterior muscle area remained significant for peak VO_2_ when adjusted for age (0.36 ± 0.2 per 1cm^2^/m^2^, *p =* 0.043) and sex (0.4 ± 0.2 per 1cm^2^/m^2^, *p =* 0.013). Paraspinal muscle area was also significantly associated with V_E_/V_CO2_ when adjusted for sex (−1.4 ± 0.6, *p =* 0.0191) and age (−1.6 ± 0.5, *p =* 0.004) (Table 6).

## 4. Discussion

In this retrospective study, we analyzed anterior and posterior muscle areas via MRI-based cross-sectional imaging in post-Fontan patients. It was feasible to perform the measurements with high reproducibility via CMR. Muscle mass measurements via CMR correlated well with peak VO_2_ and with V_E_/V_CO2_ via CPET. Larger indexed anterior and posterior SMA were associated with higher peak VO_2_, while larger paraspinal muscle area alone was associated with lower V_E_/V_CO2_.

In comparison with previous studies that analyzed indexed SMA in a healthy cohort, our population had similar results, with males having significantly higher SMA compared to their female counterparts [32]. These findings were also seen in studies that established an association between larger pectoralis muscle area with younger age and male sex in patients with acute and chronic illnesses. Refs [33,34] Posterior muscle areas at the thoracic level have not been frequently studied; therefore, it was difficult to compare our cohort to a population of healthy patients. With that said, studies that assessed posterior muscle areas at L3 did show larger SMA in males compared to females, which matched our results [32]. In this study, we established a significant relationship with increased thoracic muscle area with exercise capacity.

### 4.1. Indexed Skeletal Muscle Area and Cardiorespiratory Function

Better exercise capacity is linked to improved survival in Fontan patients [35]. In a previous study analyzing Fontan patients, peak VO_2_ was negatively associated with age, and some of the cardiorespiratory decline was attributed to natural degradation over time in Fontan physiology [36]. As expected, this trend is also seen in normal heart patients as well. In our population, age was not significantly different between high- and low-muscle groups, and the significant association of SMA to the cardiorespiratory function remained after adjusting for age. BMI was significantly lower in our high-muscle group, while the opposite was seen in our low-muscle group. This trend was likely due to a higher activity level in patients with higher muscle mass and, consequently, lower BMI. Our study supports the notion that skeletal muscle mass significantly affects exercise capacity in Fontan patients. Exercise training may increase muscle mass and improve exercise capacity [37,38,39].

We also found a significant negative association between increased posterior SMA and decreased V_E_/V_CO2_. Higher V_E_/V_CO2_ reflects ventilatory inefficiency and has been associated with worse prognosis in heart failure patients [40,41,42]. Similarly, Fernandes et al. showed an association with worse risk of mortality in Fontan patients [43]. Lower muscle mass may be synonymous with poor conditioning, resulting in a higher V_E_/V_CO2_, as seen in our study.

### 4.2. Limitations

There are several limitations to our study. Due to the lack of standardized values for the indexed SMA of anterior and posterior muscles assessed at the level of T4, we were unable to define which patients in our cohort met the clinical definition of sarcopenia. A larger cohort study with matched subjects could help address this issue and provide insight into how we can use these measurements from CMR to monitor the health of Fontan patients. In addition, when splitting our population into high- and low-muscle groups, the high-muscle group category heavily comprised males, and the low-muscle group largely comprised females. To mitigate this limitation, we analyzed SMA as a continuous variable, and the associations remained significant after adjusting for sex and age. In addition, questions concerning lifestyle habits such as daily exercise, years of exercise history, diet, and other lifestyle factors were not available in this retrospective analysis; therefore, we are unable to address other key factors contributing to body composition in this study.

## 5. Conclusions

Our study found a significant association between increased thoracic skeletal muscle mass and markers of cardiorespiratory fitness in Fontan circulation patients. This study found that skeletal muscle area, a reproducible and easily obtained measure via CMR, is positively associated with higher peak VO_2_ and negatively associated with V_E_/V_CO2_, supporting previous findings that muscle development is an important factor for exercise capacity in this population. These findings support the idea that supervised exercise activity or physical therapy could be beneficial to improving fitness in Fontan patients. In addition, with CMR muscle mass correlating well with markers of cardiorespiratory fitness, it is possible that some patients may be able to skip CPET and alternate surveillance with imaging. Moreover, imaging could potentially be used to assess response to exercise programs. More research will be needed to support this idea.

## Figures and Tables

**Table 1:** General characteristics.

Variables	N	Results
Age (y), mean ± SD	75	19.1 ± 8.6
Male sex, n (%)	75	49 (65%)
Muscle characteristics (N = 75)		
BSA-indexed anterior muscle (cm^2^/m^2^), mean ± SD		20.8 ± 5.7
BSA-indexed paraspinal muscle (cm^2^/m^2^), mean ± SD		6.5 ± 1.7
BSA-indexed total skeletal muscle (cm^2^/m^2^), mean ± SD		27.3 ± 6.6
Fontan type (N = 75)		
Atriopulmonary and other, n (%)		1 (1.3%)
Lateral tunnel, n (%)		23 (31%)
Extracardiac, n (%)		49 (65%)
Unkown, n (%)		2 (2.6%)
Ventricular Morphology (N = 75)		
Left, n (%)		35 (47%)
Right, n (%)		28 (37%)
Both, n (%)		12 (16%)

BSA—body surface area.

**Table 2:** General exercise and CMR data.

Variables	N	Results
Exercise parameters		
Peak VO_2_ (mL/kg/min)	38	27.1 ± 6.6
Peak O_2_ pulse (mL/beat), mean ± SD	19	9.6 ± 2.7
Peak HR (beats/min), mean ± SD	51	165.7 ± 25.5
% Predicted peak HR	45	83.1 ± 12.2
Peak RER	43	1.2 ± 0.1
V_E_/V_CO2_	42	36.5 ± 6.0
CMR Ventricular Volumetry		
EF (%), mean ± SD	75	49.4 ± 9.6
BSA-indexed SVEDV (mL/m^2^), mean ± SD	75	100.6 ± 41.0
BSA-indexed SVESV (mL/m^2^), mean ± SD	75	50.7 ± 21.7
BSA-indexed ventricle mass, mean ± SD	41	43.8 ± 17.2

VO_2_—oxygen consumption; BSA—body surface area; RER—respiratory exchange ratio; V_E_—minute ventilation; V_CO2_—carbon dioxide production; EF—ejection fraction; SVEDV—end diastolic volume; SVESV—end systolic volume.

**Table 3:** Sex-specific characteristics. *T*-testing was used for sample sizes >30, for those <30, Mann–Whitney U was used. Effect sizes were calculated by using Cohen’s d test for groups with similar sample sizes or sample sizes > 20. Hedges’ g test was used to calculate effect size for groups with largely different sample sizes or sizes < 20.

Variables	Female	Male	*p*-Value Effect Size (95% CI)
	Mean ± SD (N) [1st Quartile, Median, 3rd Quartile]	Mean ± SD (N) [1st Quartile, Median, 3rd Quartile]	
Age (years)	17.2 ± 7.0 (20) [13.3, 15, 21.5]	17.8 ± 7.1 (19) [12, 17, 22]	0.499 0.085 (−0.39, 0.56)
Sex (N, %)	Male: 8 (40%) Female: 12 (60%)	Male: 18 (95%) Female: 1 (5%)	0.0003 *
Height (cm)	158.5 ± 17.0 (20) [155.5, 162.9, 168]	154.6 ± 26.1 (19) [130, 165, 174.4]	0.704 0.28 (−0.20, 0.75)
Weight (kg)	65.0 ± 20.0 (20) [54.4, 68.8, 77.8]	54.7 ± 25.4 (19) [28, 59.1, 70.4]	0.136 0.24 (−0.24, 0.72)
BMI (kg/m^2^)	25.1 ± 5.3 (20) [21.1, 25.4, 28]	21.3 ± 4.9 (19) [17.1, 20.8, 24.9]	0.027 * 0.32 (−0.44, 0.51)
Anterior Wall Muscle Area (cm^2^)	25.8 ± 7.0 (20) [23, 25.6, 31.4]	41.4 ± 15.5 (19) [25.2, 42.8, 52.8]	0.002 * 0.86 (0.36, 1.35)
Paraspinal Wall Muscle Area (cm^2^)	8.7 ± 2.0 (20) [7.7, 9.3, 9.8]	12.1 ± 3.8 (19) [9.6, 11.9, 14.3]	0.002 * 0.77 (0.28, 1.26)
Peak VO_2_ (mL/kg/min)	23.8 ± 4.7 (12) [20.7, 23.4, 27.6]	32.2 ± 8.5 (9) [25.2, 36.1, 37]	0.009 * 0.15 (−0.48, 0.78)
Peak O_2_ Pulse (mL/beat)	8.1 ± 0.9 (5) [7.3, 8, 8.9]	11.1 ± 3.6 (5) [8, 12.2, 13.7]	0.144 0.83 (−0.10, 1.82)
Peak HR (beats/min)	167.3 ± 21.4 (14) [154.5, 171, 181]	173.7 ± 12.0 (10) [163.5, 173, 185.3]	0.519 0.07 (−0.51, 0.65)
% Predicted Peak HR (%)	82.8 ± 8.1 (13) [78.5, 84, 87.5]	88.6 ± 5.2 (8) [83.5, 88, 94]	0.103 0.02 (−0.61, 0.66)
Peak RER	1.1 ± 0.04 (12) [1.1, 1.1, 1.2]	1.2 ± 0.1 (9) [1.2, 1.2, 1.3]	0.006 * 0.51 (−0.14, 1.16)
V_E_/V_CO2_	40.2 ± 6.2 (11) [35, 39, 45]	32.9 ± 3.6 (9) [30.5, 33, 36]	0.006 * −0.55 (−1.21, 0.12)

BSA—body surface area; VO_2_—oxygen consumption; V_E_—minute ventilation; V_CO2_—carbon dioxide production. * Denotes *p*-value < 0.05.

**Table 4:** Anterior and paraspinal muscle correlations.

Variable	N	Indexed Anterior Muscle		Indexed Paraspinal Muscle		Indexed Total Muscle	
		r	*p*-Value	r	*p*-Value	r	*p*-Value
Peak VO_2_ (mL/kg/min)	38	0.3364	0.039 *	0.4303	0.007 *	0.4023	0.012 *
Peak O_2_ Pulse (mL/beat)	19	0.1339	0.123	0.1515	0.536	0.3692	0.120
Peak HR (beats/min)	45	0.1256	0.411	0.0478	0.755	0.1210	0.429
% Predicted Peak HR (%)	39	0.1969	0.230	−0.0297	0.858	0.1634	0.320
Peak RER	37	0.1297	0.444	0.2836	0.089	0.1823	0.280
V_E_/V_CO2_	36	−0.2013	0.239	−0.4450	0.006 *	−0.2865	0.090
EF (%)	75	−0.0899	0.443	−0.0212	0.857	−0.0827	0.481
Ventricle Mass (g/m^2^)	41	0.0743	0.644	−0.1936	0.225	0.0181	0.911
BSA-Indexed Ventricle Mass	41	0.0623	0.699	−0.1733	0.279	0.0023	0.988

VO_2_—oxygen consumption; BSA—body surface area; RER—respiratory exchange ratio; V_E_—minute ventilation; V_CO2_—carbon dioxide production; EF—ejection fraction. * Denotes *p*-value < 0.05.

**Table 5:** Low and high muscle mass characteristics. Mann–Whitney U was used due to sample sizes < 30. Effect sizes were calculated by using Cohen’s d test for groups with similar sample sizes or sample sizes > 20. Hedges’ g test was used to calculate effect size for groups with largely different sample sizes or sizes < 20.

Variable	Low Muscle (N = 20)	High Muscle (N = 19)	*p*-Value Effect Size (95% CI)
	Mean ± SD (N) [1st Quartile, Median, 3rd Quartile]	Mean ± SD (N) [1st Quartile, Median, 3rd Quartile]	
Age (years)	17.2 ± 7.0 (20) [13.3, 15, 21.5]	17.8 ± 7.1 (19) [12, 17, 22]	0.499 0.10 (−0.53, 0.73)
Sex (N, %)	Male: 8 (40%) Female: 12 (60%)	Male: 18 (95%) Female: 1 (5%)	0.0003 *
Height (cm)	158.5 ± 17.0 (20) [155.5, 162.9, 168]	154.6 ± 26.1 (19) [130, 165, 174.4]	0.704 −0.18 (−0.81, 0.45)
Weight (kg)	65.0 ± 20.0 (20) [54.4, 68.8, 77.8]	54.7 ± 25.4 (19) [28, 59.1, 70.4]	0.136 −0.45 (−1.08, 0.19)
BMI (kg/m^2^)	25.1 ± 5.3 (20) [21.1, 25.4, 28]	21.3 ± 4.9 (19) [17.1, 20.8, 24.9]	0.027 * −0.76 (−1.41, −0.10)
Anterior Wall Muscle Area (cm^2^)	25.8 ± 7.0 (20) [23, 25.6, 31.4]	41.4 ± 15.5 (19) [25.2, 42.8, 52.8]	0.002 * 1.31 (0.61, 2.0)
Paraspinal Wall Muscle Area (cm^2^)	8.7 ± 2.0 (20) [7.7, 9.3, 9.8]	12.1 ± 3.8 (19) [9.6, 11.9, 14.3]	0.002 * 1.14 (0.46, 1.82)
BSA-Indexed Anterior Muscle (cm^2^/m^2^)	15.4 ± 2.2 (20) [13.6, 15.7, 16.2]	27.5 ± 5.8 (19) [24.2, 26.2, 28.4]	<0.0001 * 2.77 (1.87, 3.65)
BSA-Indexed Paraspinal Muscle (cm^2^/m^2^)	5.2 ± 0.7 (20) [5, 5.2, 5.7]	8.2 ± 1.8 (19) [7, 7.8, 8.7]	<0.0001 * 2.18 (1.37, 2.97)
Exercise parameters:			
Peak VO_2_ (mL/kg/min)	23.8 ± 4.7 (12) [20.7, 23.4, 27.6]	32.2 ± 8.5 (9) [25.2, 36.1, 37]	0.009 * 1.22 (0.30, 2.13)
Peak O_2_ Pulse (mL/beat)	8.1 ± 0.9 (5) [7.3, 8, 8.9]	11.1 ± 3.6 (5) [8, 12.2, 13.7]	0.144 1.03 (−0.22, 2.23)
Peak HR (beats/min)	167.3 ± 21.4 (14) [154.5, 171, 181]	173.7 ± 12.0 (10) [163.5, 173, 185.3]	0.519 0.34 (−0.45, 1.13)
% Predicted Peak HR (%)	82.8 ± 8.1 (13) [78.5, 84, 87.5]	88.6 ± 5.2 (8) [83.5, 88, 94]	0.103 0.78 (−0.11, 1.66)
Peak RER	1.1 ± 0.04 (12) [1.1, 1.1, 1.2]	1.2 ± 0.1 (9) [1.2, 1.2, 1.3]	0.006 * 1.47 (0.50, 2.40)
V_E_/V_CO2_	40.2 ± 6.2 (11) [35, 39, 45]	32.9 ± 3.6 (9) [30.5, 33, 36]	0.006 * −1.34 (−2.27, −0.37)

BSA—body surface area; VO_2_—oxygen consumption; RER—respiratory exchange ratio; V_E_—minute ventilation; V_CO2_—carbon dioxide production. * Denotes *p*-value < 0.05.

**Table 6:** Multivariate analysis using BSA-indexed paraspinal or anterior muscle area as one of the independent variables. Each multivariable analysis included two variables as independent variables to adjust for sex then age.

	Variables	Indexed Paraspinal Muscle	Indexed Anterior Muscle	Indexed Total Muscle
		Parameter Estimate ± SD (*p*-Value)	Parameter Estimate ± SD (*p*-Value)	Parameter Estimate ± SD (*p*-Value)
Age-adjusted	Peak VO_2_	1.7 ± 0.6 (0.008) *	0.4 ± 0.2 (0.043) *	0.4 ± 0.2 (0.012) *
	V_E_/V_CO2_	−1.4 ± 0.6 (0.019) *	−0.1 ± 0.2 (0.522)	−0.1 ± 0.2 (0.522)
Sex-adjusted	Peak VO_2_	1.5 ± 0.6 (0.012) *	0.4 ± 0.2 (0.013) *	0.4 ± 0.1 (0.005) *
	V_E_/V_CO2_	−1.6 ± 0.5 (0.004) *	−0.06 ± 0.1 (0.616)	−0.2 ± 0.1 (0.115)

VO_2_—oxygen consumption; V_E_—minute ventilation; V_CO2_—carbon dioxide production. * Denotes *p*-value < 0.05.

## Data Availability

The data that support the findings of this study are available from the corresponding author upon reasonable request.

## Supplementary Materials

The following supporting information can be downloaded at: https://www.mdpi.com/article/10.3390/jcm12072689/s1, Table S1: Ventricular Morphology Exercise Parameters.
