# Supplementary material for: Surrogates of Muscle Mass on Cardiac MRI Correlate with Exercise Capacity in Patients with Fontan Circulation
Source: J Clin Med. 2023 Apr 4;12(7):2689. doi: 10.3390/jcm12072689 (PMC10095035; doi:10.3390/jcm12072689)
Supplement: Supplementary file 1 [file jcm-12-02689-s001.zip › jcm-2289671-supplementary.pdf]

Journal of
Clinical Medicine
MDPI

Supplementary Materials

Table S1. Ventricular Morphology Exercise Parameters.

Variables	LV morphology	RV Morphology	BV Morphology	
	**Mean ± SD (N)**	**Mean ± SD (N)**	**Mean ± SD (N)**	**P-Value**
**Exercise Parameters:**				
Peak VO_2 (mL/kg/min)	28.4 ± 7.3(26)	25.8 ± 6.4 (11)	27.6 ± 6.6 (7)	0.581
Peak HR (beats/min)	165.0 ± 28.9 (27)	165.5 ± 18.9 (15)	167.4 ± 19.3 (9)	0.967
% Predicted Peak HR (%)	82.4 ± 14.4 (24)	82.5 ± 8.0 (14)	85.7 ± 6.9 (7)	0.797
Peak RER	1.2 ± 0.1 (26)	1.1 ± 0.08 (11)	1.2 ± 0.1 (6)	0.223
Peak Saturation (%)	90.0 ± 5.4 (26)	91.0 ± 5.3 (15)	87.9 ± 5.1 (8)	0.431
V_E/V_{CO2}	36.2 ± 6.0 (26)	38.6 ± 4.9 (11)	37.1 ± 7.2 (5)	0.521

VO_2, oxygen consumption; RER, respiratory exchange ratio; V_E, minute ventilation; V_{CO2}, carbon dioxide production. * Denote P<0.05.
